# Medical Progress in Italy

**Published:** 1888-03

**Authors:** 


### MEDICAL PROGRESS IN ITALY.

The full report given in the November number (1887) of the *Medical Journal and Examiner*, of the last bi-ennial meeting of the Italian Medical Association, showed the strides that that regenerated country is making to regain the position it once held in the medical world. The letter, in another part of this number of the *Journal*, from our Italian correspondent, regarding medical matters in that country will be found to be interesting in several respects. It recalls the names and works of Italian physicians, surgeons, and teachers, connected in times long past with the old university of Pavia, that are as familiar to the medical students of to-day, and will be to those of future ages, as they were to the pupils of those famous men when they were making the reputation of that university.

It will be a surprise to many, who have not visited that old institution in recent years, to learn of its great awakening, and of the amount of scientific work that is again being done in it, and for the regeneration of Italy. In this work it is prominent, but it does not stand alone in the progress of medical education in that country, as well as in the higher education of Italians generally.

Whilst age has brought to the university accumulations that are of great value in study and in investigation, the disadvantage which attends many years of existence in hospitals, is strikingly shown in our correspondent's report of the rate of mortality at the clinique described. It affords another strong argument against the continuance of the use of hospitals that have long been in existence, and, in this instance, either of incomplete observance of modern antiseptic methods or of the inefficacy of those methods to secure asepsis in buildings long used for hospital purposes.

The brief outline, given by our correspondent, of the new Polyclinic in Rome, bears further evidence of the recent awakening from the lethargic state which characterized it for so many years; and for Rome, to-day, is claimed the credit of publishing the only *daily* medical journal in the world—*La Riforma Medica*, edited by Professor Gætona Rummo.

